# Special Issue “Extreme Mechanics in Multiscale Analyses of Materials”

**DOI:** 10.3390/ma16072886

**Published:** 2023-04-05

**Authors:** Bin Wang, Arash Soleiman-Fallah

**Affiliations:** 1 Department of Mechanical and Aerospace Engineering, Brunel University London, Uxbridge UB8 3PH, UK; 2 Faculty of Technology, Art and Design, Department of Mechanical, Electronics and Chemical Engineering, Oslo Metropolitan University, Pilestredet 46, 0167 Oslo, Norway

The responses and behaviour of engineering structures and materials subjected to various types of loading, particularly those under extreme loading such as earthquakes, explosions, and impacts, as well as under exposure to environmental elements, are of critical significance for the safety and integrity of said structures to fulfil their intended functions. As academics involved in research on and the development of extreme mechanics, multiscale simulation, and constitutive formulation and modelling, we welcomed the opportunity granted by MDPI’s journal *Materials* to allow us act as the guest editors of this Special Issue entitled, “Extreme Mechanics in Multiscale Analyses of Materials”. This Special Issue has been developed to provide a platform for world-wide researchers studying material behaviour in extreme loading conditions using multiscale analyses, numerically or experimentally, to disseminate their research outcomes, and it includes a collection of high-quality original research works. The Special Issue has received a significant amount of attention, and it attracted a great number of submissions, from which 12 were accepted for publication.

The papers included in this Special Issue deal with a broad spectrum of interests in analytical, computational, and experimental studies on extreme types of material behaviour. The included papers encompass many areas of extreme mechanics modelling including: a strength investigation using detailed microfractography analysis of fractures formed during static tensile tests of steel Armstal 550 [1], a study on the effect of NTO content on the properties of an HMX-based cast-PBX (polymer bonded explosive) [2], the dependency of contact length on cutting speed and other variables performed by the optical method in cutting processes [3], bone molecular models at the nanoscale [4], molecular models of bones at the nanoscale, the characterisation of composites’ mechanical behaviour at low temperatures [5], the diffusion of hydrogen atoms through the grain boundaries of materials, the failure properties of batteries under axial forces [6], the deformation and failure properties of Ni lithium batteries [7], the effect of material heterogeneity on the environmentally assisted cracking growth rate of Alloy 600 for safe-end welded joints [8], the high strain yielding of the additive manufacturing of Inconel 625 through laser melting [9], the effect of the yield strength distribution of welded joints on the crack propagation path and the crack mechanical tip field [10], the effect of mechanical heterogeneity on strain and stress fields at crack tips of SCC in dissimilar metal welded joints [11], and the characterisation of mechanical heterogeneity in dissimilar metal welded joints [12].

We would like to thank all of the authors who contributed to this Special Issue and all of the reviewers who took time and efforts selflessly to provide comments and opinions, including suggestions and advice on the submitted work to ensure the quality and integrity of the selection process and the papers selected. In addition, we would like to thank all of the staff from the *Materials* editorial office for their great support in making this Special Issue possible. Special acknowledgment must also go to the section managing editor, Ms. Lili Li, for her continuous support during the preparation and organisation of this Special Issue. 
